# Dipeptidyl-Peptidase-4 and Glucagon-like-Peptide-1, a Link in the Connection between Periodontitis and Diabetes Mellitus—What Do We Know So Far?—A Scoping Review

**DOI:** 10.3390/jcm13030903

**Published:** 2024-02-04

**Authors:** Theodora Claudia Gheonea, Petra Șurlin, Flavia Mirela Nicolae, Dorin Nicolae Gheorghe, Dora Maria Popescu, Ion Rogoveanu

**Affiliations:** 1 Center for IBD Patients, Faculty of Medicine, University of Medicine and Pharmacy of Craiova, 200345 Craiova, Romania; 2 Department of Periodontology, Research Center of Periodontal-Systemic Interactions, Faculty of Dental Medicine, University of Medicine and Pharmacy of Craiova, 200349 Craiova, Romaniapopescudoramaria@yahoo.com (D.M.P.); 3 Department of Gastroenterology, Faculty of Medicine, University of Medicine and Pharmacy of Craiova, 200349 Craiova, Romania

**Keywords:** periodontitis, diabetes mellitus, dpp-4, glp-1, *Porphyromonas gingivalis*

## Abstract

Periodontitis is a common condition affecting the tissues surrounding and supporting teeth. In addition to oral health concerns, periodontal disease increases the chance of developing systemic illnesses including type 2 diabetes mellitus. *Porphyromonas gingivalis,* a key-stone pathogen that has been linked to the pathophysiology of periodontal disease, can generate a series of dipeptide producing exopeptidases, dipeptidyl peptidases (DPP). DPP-4 levels in gingival crevicular fluid have been shown to increase during active periodontal disease, which may lead to their association with the disease’s progression. Following oral glucose administration, mice injected with DPP-4 had higher blood glucose than the control group. DPP-4 inhibitors are used to treat patients with type 2 diabetes mellitus in order to extend the half-life of incretins. Elevated glucagon-like peptide-1 (GLP-1) levels following periodontal therapy could be considered new and applicable real-world evidence confirming the experimental findings of a beneficial interaction between oral microbiota and incretin axis. GLP-1 receptor agonist exendin-4 enhanced the osteoblast proliferation and development of these stem cells and inhibited the effects of glucose on the cells. In addition to lowering blood sugar, liraglutide, a GLP-1 receptor agonist, also possesses anti-inflammatory and bone-protective properties. These findings support the use of GLP-1 in the management and prevention of diabetic periodontitis.

## 1. Introduction

Periodontitis is a common condition affecting the tissues surrounding and supporting teeth in the oral cavity. It affects 20% to 50% of the global population and lowers overall quality of life [1]. It is a persistent inflammation primarily brought on by a complex of endogenous anaerobic rods that proliferate in subgingival pockets, thus forming subgingival disbiotic dental plaque [2]. These anaerobes are thought to form commensal communities in subgingival pockets, where they mutually support each other’s growth and survival in specific conditions [3]. The three main periodontopathic bacteria related to severe forms of periodontal disease are *Porphyromonas gingivalis*, *Tannerella forsythia*, and *Treponema denticola* [4].

The anaerobic bacteria *P. gingivalis* is directly linked to inflammatory alveolar bone resorption [5]. In terms of nutrition, *P. gingivalis* is an asaccharolytic bacteria, meaning that its only source of carbon and energy are amino acids, which appears to be beneficial for its colonization in subgingival plaque, as most of the other bacteria favor sugar [6,7]. Porphyromonas species also primarily incorporate amino acids as dipeptides [8]. Furthermore, regarding its development and virulence, this bacterium can generate proteolytic enzymes, such as those that degrade collagen, elastase-like enzymes, trypsin-like proteases, aminopeptidases, and a series of dipeptide producing exopeptidases, dipeptidyl peptidases [9,10,11]. These proteolytic enzymes, such as dipeptidyl peptidase 4 (DPP-4), which aids in the disintegration of collagen and furthermore prevents fibronectin and gingival fibroblasts from binding, are thought to be essential in the periodontium’s destruction [9,12]. *P. gingivalis* DPP 4 also binds to fibronectin, regulating bacterial adherence to host cells, and hydrolyzes biologically active peptides such as substance P, fibrin inhibitory peptide, and casomorphin. Additionally, using a mouse subcutaneous abscess model, it was showed that DPP4 was related directly to biofilm development [12].

Human DPP4 is a multifunctional molecule that is involved in the breakdown of a wide variety of bioactive proteins and peptides as well as T-cell activation [7]. DPP-4 has been suggested as a prognostic or diagnostic marker for numerous kinds of cancers, infection with viruses, hematologic malignancies and illnesses related to the immuno-inflammatory system [9,13]. Because of their early clinical significance, autoantibodies against DPP-4 may be investigated as biomarkers in various research, with the possibility that they contribute to the pathophysiology of rheumatoid arthritis [13].

While DPP-4 can be found across numerous eukaryotes, it was believed to be exclusive to *P. gingivalis* and *Chryseobacterium* in bacteria [9]. Later, the expression of DPP4 in *T. forsythia* and *P. intermedia*, which had enzymatic characteristics similar to those of *P. gingivalis*, was first shown in research by Ohara-Nemoto et al. in 2017 [14]. Despite having a modest amino acid similarity (23.8%) with human DPP4, *P. gingivalis* DPP4 demonstrates substrate preference similar to that of human DPP4 and a very similar overall structure to human isoform [6].

By breaking down incretin peptides including glucagon-like peptide 1 (GLP-1) and glucose-dependent insulinotropic polypeptide, which stimulate the release of insulin from pancreatic β cells, DPP4 plays a crucial role in regulating postprandial hyperglycemia [6,7].

In addition to oral health concerns, some studies associate periodontal disease with systemic illnesses including type 2 diabetes mellitus, cardiovascular diseases, Alzheimer’s disease, obesity, adverse pregnancy outcomes, rheumatoid arthritis, osteoporosis and cancer [13,15,16]. There is a complicated bidirectional association between diabetes mellitus and periodontitis which comprises pro-inflammatory mediators and hereditary factors. The presence of periodontitis may exacerbate insulin resistance, a condition characterized by hyperglycemia and the production of advanced glycation end products [16,17]. Advanced glycation end products consequently result in an excess of IL-6, IL-1, and TNF-α (tumor necrosis factor alpha). Advanced glycation end products therefore contribute to both an increased neutrophil response to periodontal bacteria and poor wound healing (inappropriate collagen turnover in fibroblasts) [16,17,18]. Patients who have both periodontitis and type 2 diabetes also have a significantly higher overall cardiovascular disease death rate [19,20].

*Aim:* Since it has been established that DPP-4 and GLP-1 are important incretins linked to the pathogenicity of diabetes mellitus, with their involvement in periodontal disease having been proven, the present review aims to update the recent data regarding these incretins’ roles in patients with periodontitis and diabetes, with an emphasis on patients who have both diabetes mellitus and periodontitis. It aims to draw attention to the fact that there are sufficient data to suggest that both illnesses and their treatments may be related through these two compounds.

## 2. Materials and Methods

### 2.1. Search Strategy

Two independent researchers conducted a database inquiry spanning from November to December 2023, including data up until October 2023. The search encompassed pivotal scientific databases such as Medline (accessible via PubMed). The keywords utilized throughout this investigation were as follows: “dpp4”, “dpp-4”, “glp-1”, “glp1”, “periodontal”, “periodontitis “, and “diabetes mellitus”.

### 2.2. Inclusion and Exclusion Criteria for the Selected Studies

The incorporated studies were required to fulfill specific prerequisites: comprehensive publication, availability in full-text, and composition in the English language. The criteria for exclusion from the research findings encompassed self-reported studies, letters, editorials, and abstracts. The studies selected for inclusion underwent subsequent evaluation, were archived using Zotero software (Version 6.0.29) (Fairfax City, VA, USA), and duplicate entries were eliminated. Out of the total 32 identified studies, 2 were excluded due to their sole availability in the Chinese language.

### 2.3. Information Structuring and Review Writing

The extracted data were structured into two parts: (i) interactions of DPP-4, GLP-1 and periodontitis; and (ii) interactions of DPP-4, GLP-1 in diabetes and periodontitis.

## 3. Periodontal Perspective on DPP-4 and GLP-1′s Actions

Previous research has demonstrated that single nucleotide polymorphisms in the interleukin 1 gene, interleukin 10 gene, Fc gamma gene, and tumor necrosis factor-alpha gene are related to periodontitis in terms of genomic indicators of the disease. Specific genes, including DPP-4, showed statistically significant differences between the healthy volunteers and patients with severe periodontitis. Those genes may include single nucleotide polymorphisms that serve as genetic markers for severe periodontitis, according to these findings [21].

There has been evidence since 1995 that both DPP-2 and DPP-4 levels in gingival crevicular fluid have been shown to increase during active disease, which may lead to their association with the progression of periodontal disease. The gingival crevicular fluid DPP-2 and DPP-4 activity has been shown in this study to be a strong predictor of periodontal attachment loss in the future [22].

While there was no significant difference in the DPP-4 activities between the groups with localized and generalized chronic periodontitis, there was a substantial increase in the enzyme activities of both groups when compared to the periodontally healthy group. Subjects with periodontitis (76.3–91.7%) and those in periodontally healthy conditions (5%) both exhibited *P. gingivalis*. The prevalence of *P. gingivalis* and all clinical indicators showed a positive association with DPP-4 activity. The study concluded that *P. gingivalis* and periodontitis are associated with DPP-4 activity but not alanine aminopeptidase’s activity [9]. According to earlier research, individuals with gingivitis or periodontitis had higher DPP4 values when their clinical parameters increased [9,23,24].

In a recent study, Jiang added various amounts of *P. gingivalis* to microcosm biofilms that were cultivated using the pooled saliva of healthy individuals. In all biofilms, he discovered that DPP-4 activity significantly increased on day 10 in comparison to day 3. Nonetheless, compared to biofilms without the bacterium, those containing *P. gingivalis* exhibited much greater activity [25]. DPP4 activity may be seen in a variety of bacterial species. A dysbiotic state may have been induced in the microcosm biofilm composition by the growing condition (serum in the growth medium, for example), which encouraged the development of DPP4-producing bacteria, thus promoting the development of biofilms and the colonization of mixed species in subgingival plaque. Because of this, these two biomarkers may be utilized to identify the dysbiotic condition of the microbiota in vitro, but not the quantity of *P. gingivalis* in biofilms [12,25].

As an enzyme and membrane receptor composed of 723 amino-acids [12], DPP-4 has been found to have a variety of applications. When co-expressed with other enzymes, these enzymes may function together to perform various functions. This allows other enzymes to take over and complete DPP-4 tasks in its lieu. Therefore, additional enzymes could have been involved and prevented the improvement in clinical periodontal parameters even in the presence of DDP-4 inhibition, as demonstrated by the ELISA test [26].

Compared to mice challenged with a DPP-4-deficient strain, mice injected with the *P. gingivalis* W83 strain were seen to pass away and to develop abscesses more frequently. But rather than pathogenicity potential, this event could indicate DPP-4′s nutritional value [6,27].

One of the main characteristics of chronic periodontitis is the breakdown of periodontal tissues. Glycine (Gly)-proline (Pro)-X makes up the majority of type I collagen, which is one of the primary constituents of periodontal tissues. The N-terminal ends of polypeptide chains are where the serine protease DPP-4 cleaves the X-Pro or X-alanine dipeptide [9,26].

It is possible that *P. gingivalis*, a keystone pathogen that has been linked to the pathophysiology of periodontal disease, and host tissues are the main sources of DPP-4 in saliva [9,28]. When considered as a whole, the extracellular protein degradation process in *P. gingivalis* is started by gingipains and ultimately reaches dipeptides mediated by DPPs with the assistance of acylpeptidyl-oligopeptidase and protyl tripeptidyl-peptidase A [8]. Nutritional polypeptides should be split apart initially into oligopeptides by endopeptidases and Lys- and Arg-gingipains at the outer membrane, and then into dipeptides by DPP-4, DPP-5, DPP-7, and DPP-11, as nutritional polypeptides are mostly integrated as dipeptides in bacterial cells [6,7]. The synthesis of energy within the bacterium is dependent upon the metabolism of amino acids, which are mostly integrated as dipeptides by the action of the proton-dependent oligopeptide transporter [12]. In the periplasmic area, *P. gingivalis* DPP-4, DPP-5, DPP-7, and DPP-11 were found. These are thought to be appropriate for full dipeptide synthesis [28]. DPP-4 appears to be involved in the periplasmic degradation of extracellular proteins and is concentrated in outer membrane vesicles in heme excess circumstances [6].

When compared to type 2 diabetes mellitus patients without periodontitis, gastric inhibitory polypeptide (GIP) and GLP-1 were shown to be elevated, and GIP was associated with a diagnosis of periodontitis, which is defined as a pocket depth greater than 4 mm. Also, a weakly positive association was found between the level of GLP-1 and glucagon and the number of sites with PD > 4 mm [20,29].

GLP-1 was directly correlated with the full-mouth bleeding score, mean clinical attachment levels, and periodontal pocket depth in a study involving both obese and non-obese subjects. A different noteworthy and innovative finding is that, in the presence of periodontitis, the GLP-1 levels of non-obese and obese people are comparable and are associated with the degree of periodontal inflammation. This finding suggests that oral inflammation plays a more significant role in influencing the incretin axis than obesity status does [30].

A clinical study published in 2019 revealed, for the first time, that obese non-diabetic individuals with periodontitis had a compromised incretin axis in addition to a relative hyperglucagonemia. These findings may worsen the patients’ glucose tolerance and help to explain certain aspects of their higher risk of developing diabetes. It was discovered that glucagon levels were elevated and were linked with the severity of the condition, but GLP-1 levels were substantially decreased. It was hypothesized that the oral/gut microbiota could have an active role in determining this clinical state and hasten the breakdown of glucose homoeostasis. According to their research, individuals with periodontitis may benefit from a novel critical target for preventing the development of type 2 diabetes: the incretin axis [31].

The pattern of glucoregulatory hormones in obese and nonobese individuals can be enhanced by treating periodontitis, as demonstrated in a recent study. This improvement is primarily due to an increase in GLP-1 and GIP, and it is also noticeable over a different time frame in the obese compared to the nonobese. The distinctive feature of these preliminary results is that the changes in glucoregulatory hormones occurred without any detectable modifications to the metabolic phenotype of the individuals under investigation; there was no reported decrease in weight, lipid profile, or blood pressure measurements [30]. According to one in vitro study, advanced glycation end products’ effects on osteogenic potential might be mitigated by GLP-1. Furthermore, in human periodontal ligament stem cells, the mechanism of GLP-1 suppresses the adverse effects of advanced glycation end products, presumably through the inhibition of PKCβ2 phosphorylation [32].

Due to its immediate inactivation by the commonly found proteolytic enzyme DPP-4, bioactive GLP-1 in circulation has a half-life of around two minutes. Consequently, GLP-1 analogs, which can activate GLP-1R and have a substantially longer half-life, are frequently included in modern glp-1 medications [32].

In vitro research using periodontal ligament stem cells revealed that the GLP-1 receptor agonist exendin-4 both enhanced the osteoblast proliferation and development of these stem cells and inhibited the effects of glucose on the cells [33,34]. Rats with ovariectomy-induced osteoporosis have showed improved bone strength, reduced trabecular bone degradation, and increased evidence of bone formation when treated with the GLP-1 receptor agonist exendin-4 [35]. These findings indicate that these medications could be used in diabetic individuals suffering from periodontitis [20,36]. Additional research revealed that a high glucose environment impeded the osteogenesis of periodontal ligament stem cells. However, exendin-4 mitigated this inhibitory effect by influencing the MAPK and WNT signaling pathways, which are implicated in osteoblast differentiation and bone formation [36].

A GLP-1 receptor agonist called liraglutide (LIRA) was shown to have an impact on the progression of periodontitis. Rats with ligature-induced periodontitis showed increased expression of inflammatory genes in the gingiva, infiltration of inflammatory cells, and loss of alveolar bone, all of which were ameliorated by liraglutide. Liraglutide was also shown to suppress inflammatory M1 macrophages associated with periodontitis in the gingiva and to reduce alveolar bone loss by decreasing osteoclast production. Their findings emphasized the GLP-1 receptor’s direct impact on periodontitis therapy, instead of its effects on decreasing blood sugar and preventing obesity [37].

Human periodontal ligament cells were stimulated with lipopolysaccharide (LPS) in order to resemble periodontitis in vitro. LIRA stimulated the cells’ migration, proliferation, and osteogenic differentiation while inhibiting the inflammatory response in a time-dependent approach. ALP and Runx2 expressions were raised, mineralization node formation was increased, and TNF-α and IL-6 production were suppressed by LIRA therapy [38]. Other authors also showed that following LIRA therapy, there was an increase in the production of mineralized nodules. Additionally, *P. gingivalis*-LPS decreased the osteogenesis of human periodontal ligament cells and significantly promoted the Wnt/β-catenin signaling pathway. Nevertheless, LIRA reversed these effects. The in vivo findings demonstrated that LIRA administration decreased alveolar bone resorption, lowered TNF-α, IL-1β, and IL-6 concentrations, and lessened inflammatory cell infiltration in periodontal tissues [39].

In human dental pulp-derived stem cells, liraglutide peptide administration enhanced osteoblast development by increased calcium deposition, ALP activity, and osteoblast marker genes, Runx2, type 1 collagen, osteonectin, and osteocalcin. Furthermore, liraglutide enhanced calcium deposition, osteoblastic cell count, collagen 1α, osteonectin, osteocalcin, Runx2a, and Ca/P ratio in scales in an in vivo research study using a zebrafish scale regeneration model. Overall, in human dental pulp-derived stem cells, GLP-1R activation improves osteoblast development via Runx2/LncRNA-LINC00968/miR-3658 signaling, and in zebrafish scale regeneration, it stimulates bone production [40].

A recent paper examined the therapeutic effect of LIRA on experimental periodontitis in rats with diabetes. Alveolar bone resorption was reduced, alveolar bone microstructure was enhanced, and periodontal inflammation and damage were minimized with LIRA treatment. In addition, LIRA decreased blood glucose levels and prevented serum IL-6, TNF-α, and IL-1β from being released. Furthermore, following LIRA administration, there was a decrease in the RANKL/OPG ratio. The scientists concluded that, in diabetes-associated periodontitis, LIRA not only regulates blood glucose levels but also lessens inflammation and bone loss while promoting osteogenic differentiation. These findings suggest that LIRA might be employed as an additional treatment for diabetes-periodontitis comorbidity [41].

## 4. Implications of DPP-4 and GLP-1 in the Association between Diabetes and Periodontitis

Periodontopathic gram negative bacteria’s lipopolysaccharide produces an increased level of tumor necrosis factor-α, which proceeds to migrate from the liver to the rest of the body and causes insulin resistance. This chronic inflammation currently explains the two-way relationship between periodontal disease and type 2 diabetes mellitus [6]. Evidence from several meta-analyses of randomized clinical trials showing improvement in HbA1c levels of around 0.4–0.7% after periodontal therapy in individuals with combined periodontitis and type 2 diabetes mellitus further supports the association between periodontitis and diabetes [20].

Hyperglycemia, a result of insufficient insulin synthesis or activity, is a hallmark of diabetes mellitus, an inflammatory illness. Two intestinal hormones produced following meal consumption, glucose-dependent insulinotropic polypeptide (GIP) and glucagon-like peptide-1 (GLP-1), partially regulate the production of insulin and glucagon in people with normal blood sugar levels. Once released, the body’s widely distributed enzyme dipeptidyl peptidase-4 (DPP-4) quickly inactivates GIP and GLP-1, sometimes referred to as incretins [26]. The breakdown of incretins by the periodontopathic bacterium DPP-4 has been suggested as a further and direct relationship between the two disorders, through an impairment or the glycemic control [6,20]. DPP-4 is responsible for the release of His/Thr-Ala from the N terminus of incretin, which leads to the inactivation of GLP-1 and GIP. DPP-4 inhibitors are used to treat patients with type 2 diabetes mellitus in order to extend the half-life of incretins, as active incretins cause pancreatic β cells to secrete insulin. Researchers hypothesized a connection between bacterial DPPs and degradation of bioactive peptides that cause disruption of homeostasis or physiological regulatory systems in humans after discovering that DPP-4 of *P. gingivalis* and other periodontopathic bacteria degrades incretins and modifies host blood glucose levels [7].

Following oral glucose administration, mice intravenously injected with DPP-4 had significantly higher blood glucose than the control group. This was followed by a reduction in plasma active GLP-1 and insulin levels. Hence, bacterial DPP-4 could reduce the host’s incretin concentrations when these microorganisms enter the circulation through routine activities (recurrent bacteremia). Through molecular processes of bacterial DPP-4 activities, periodontopathic bacteremia could exacerbate diabetes mellitus. These results were the first to demonstrate that periodontopathic bacterial DPP4 is capable of altering blood glucose levels in the same way as mammalian DPP-4 [14].

The multifunctional nature of incretins makes them prospective targets for periodontopathic bacterial DPPs, which may modify human homeostasis by inactivating gastrointestinal hormones, neuropeptides, and chemokines, among other bioactive peptides [6,7]. GLP-1 receptor (GLP-1R) agonists and DPP-4 inhibitors are two examples of novel diabetes medications that work to enhance the function of incretins because of their critical role in glycemia regulation. In this case, the Food and Drug Administration has authorized the medications exenatide (a GLP-1R agonist) and sitagliptin (a DPP-4 inhibitor) for clinical use in diabetic patients [26]. Exendin-4 is being employed extensively in the therapeutic treatment of type 2 diabetes because it has a biological effect that is comparable to that of GLP-1 in vivo but has a longer half-life and a lesser tendency to degrade [36]. The rapidly occurring inactivation of GLP-1 and GIP is caused by the cleavage of the peptide link between the second Ala and third Glu by human DPP-4. DPP-4 inhibitors are therefore often used to treat people with type 2 diabetes. It is reasonable to assume that periodontopathic bacterial DPP-4 is involved in the regulation of blood glucose levels due to the shared enzymatic characteristics [6,7].

DPP-4 inhibitors are a type of oral antidiabetic drugs that suppress incretin breakdown and enhance insulin production and glucose tolerance. Examples of these drugs include sitagliptin, vildagliptin, and allopurin [6]. The periodontopathic bacterial DPP-4 is likewise inhibited by human DPP-4 inhibitors such as P32/98, vildagliptin, and sitagliptin [6]. Nevertheless, other authors reported that for type 2 diabetes patients which are administered DPP-4 inhibitors, these medications ineffectively prevent *P. gingivalis* DPP-4 from entering the circulation [10]. Other authors reported that the rats’ alveolar bone loss and collagen degradation could not be stabilized or reduced by exenatide, a GLP-1 agonist, or by sitagliptin, a DPP-4 inhibitor, even if the expression of Il1β, Nos2, and Mmp9 was reduced [26]. In contrast, recent studies report otherwise. One specific marker of early osteoblast development is the high activity and expression of the enzyme ALP, which is released by osteoblasts. Osx is a transcription factor limited to osteoblasts that is required for the forming of bones, and Runx2 is one of the earliest and most specific marker genes involved in bone formation. Exendin-4, a GLP1 receptor agonist, elevated the phosphorylation of P38, JNK, and ERK1/2 as well as the osteogenesis-related gene expression of ALP, Runx2, and Osx. Additionally, in normal or high glucose settings, the levels of p-GSK3β, Runx2, LEF, and total β-catenin increased following exendin-4 therapy [36].

Notably, bacterial DPPs may be implicated in host physiological activities in addition to producing dipeptides for bacterial nutrition, habitat segregation, and adaptability [11]. The N-terminal dipeptide from the incretin peptide hormones GLP-1 and GIP is cleaved by DPP-4 from *P. gingivalis*, *T. forsythia*, and *Prevotella intermedia* in a mouse model in a manner similar to that of human DPP-4 [14]. Both human and periodopathic bacterial DPP-4 break down incretin and alter mice’s blood glucose levels [6]. As a result of incretins’ ability to increase insulin production from pancreatic β cells following eating, blood insulin levels were lowered, and postprandial hyperglycemia was subsequently enhanced when bacterial DPP-4 restricted the proteolysis of incretins. These results clearly suggested that periodontopathic bacterial DPP-4 is also involved in type 2 diabetes patients’ glucose control [11].

GLP-1 and GIP were discovered to be effectively degraded by *P. gingivalis* cells. In an assessment of glucose tolerance, recombinant DPP-4 from *P. gingivalis* was injected intravenously into mice. This resulted in a decrease in insulin and plasma GLP-1 active form. Additionally, there was a significant increase in postprandial hyperglycemia and a delay in the reduction of blood glucose levels. Because dental procedures, toothbrushing, and mastication can result in oral bacteremia, people with severe periodontal disease are prone to experience periodontopathic bacteremia. This can occur considering bacterial DPP-4 in the bloodstream breaks down incretins, aggravating type 2 diabetes mellitus [6,7].

*P. gingivalis* DPP-4 and human DPP-4 have a very similar substrate specificity and overall structure [20]. However, it was shown that the clinically utilized DPP-4 inhibitors had limited affinity for bacterial DPP-4 after screening the existing inhibitor collection and docking compounds in the *P. gingivalis* DPP-4 active site. However, there is some overlap between the *P. gingivalis* DPP-4 and DPP-9 pharmacophores [10]. The discovery of periodontopathic DPP-4-specific inhibitors and their use in patients with type 2 diabetes mellitus could work in conjunction to lower blood glucose levels since human DPP-4 inhibitors are less effective against bacterial DPP-4 [6].

## 5. Future Perspectives

Regardless of the existence of diabetes, GIP and GLP-1 may be interesting factors in the metabolic dysregulation of individuals with periodontitis; in fact, GIP encourages bone growth and periodontal bacteria contribute to the breakdown of human incretins [30]. Considering this particular setting, hypoglycemic medications that have anti-inflammatory properties or bone-tissue protecting properties may prove beneficial in restraining the advancement of periodontitis. Since these medications are currently being used at large dosages to treat obesity, regardless of the existence of type 2 diabetes, this concept is especially relevant for overweight people [30,36]. Consequently, elevated GLP-1 and GIP levels following periodontal therapy could be considered new and applicable real-world evidence confirming experimental findings of a beneficial interaction between oral microbiota and the incretin axis, which is more likely to be related to the direct mouth-gut crosstalk as an anatomical and functional continuum rather than mediated by alterations in systemic inflammation [30]. In vitro, GLP-1 effectively restored damage that was caused by advanced glycation end products to human periodontal ligament stem cells during osteogenesis. These findings support the use of GLP-1 in the management and prevention of diabetic periodontitis [32].

Human systemic disorders are intimately associated with bacterial DPP-4. Due to their strong affinity for multifunctional incretin peptides and other bioactive peptides such as neuropeptides, chemokines, and gastrointestinal hormones, periodontopathic bacterial DPPs play an essential function in regulating human homeostasis through their breakdown [6]. Proteins implicated in the synthesis and integration of dipeptides are becoming important options in the treatment of systemic illnesses associated with periodontal disease [6]. Patients with mild to moderate type 2 diabetes must use antidiabetic medications, such as thiazolidinediones, biguanides, and sulfonylureas, to regulate their blood sugar levels. Limiting the number of medicines required for both conditions might be beneficial if these therapies exhibit a beneficial impact on periodontitis. Recently, LIRA received approval for the treatment of diabetes. In addition to lowering blood sugar, LIRA also possesses anti-inflammatory and bone-protective properties [38,39].

## 6. Conclusions

In conclusion, this study highlights the potential interconnections between periodontitis and diabetes mellitus, through the action of DPP-4 and GLP-1. Moreover, the findings suggest that medications used to manage type 2 diabetes might offer ancillary benefits in mitigating or influencing the course of periodontitis. Further exploration and targeted research is warranted to elucidate the specific mechanisms and therapeutic implications, fostering a more comprehensive understanding of the intricate relationship between diabetes management, medication regimes, and periodontal health.

## Data Availability

Data used to support the findings of this study are available from the corresponding author upon request.

## References

[1] Sanz M., Herrera D., Kebschull M., Chapple I., Jepsen S., Beglundh T., Sculean A., Tonetti M.S., EFP Workshop Participants and Methodological Consultants (2020). Treatment of stage I–III periodontitis—The EFP S3 level clinical practice guideline. J. Clin. Periodontol..

[2] Rosier B.T., Marsh P.D., Mira A. (2018). Resilience of the Oral Microbiota in Health: Mechanisms That Prevent Dysbiosis. J. Dent. Res..

[3] Kilian M., Chapple I.L., Hannig M., Marsh P.D., Meuric V., Pedersen A.M., Tonetti M.S., Wade W.G., Zaura E. (2016). The oral microbiome—An update for oral healthcare professionals. Br. Dent. J..

[4] Hajishengallis G., Lamont R.J. (2012). Beyond the red complex and into more complexity: The polymicrobial synergy and dysbiosis (PSD) model of periodontal disease etiology. Mol. Oral Microbiol..

[5] Mysak J., Podzimek S., Sommerova P., Lyuya-Mi Y., Bartova J., Janatova T., Prochazkova J., Duskova J. (2014). Porphyromonas gingivalis: Major periodontopathic pathogen overview. J. Immunol. Res..

[6] Nemoto T.K., Ohara Nemoto Y. (2021). Dipeptidyl-peptidases: Key enzymes producing entry forms of extracellular proteins in asaccharolytic periodontopathic bacterium *Porphyromonas gingivalis*. Mol. Oral Microbiol..

[7] Ohara-Nemoto Y., Shimoyama Y., Ono T., Sarwar M.T., Nakasato M., Sasaki M., Nemoto T.K. (2022). Expanded substrate specificity supported by P1′ and P2′ residues enables bacterial dipeptidyl-peptidase 7 to degrade bioactive peptides. J. Biol. Chem..

[8] Ohara-Nemoto Y., Rouf S.M., Naito M., Yanase A., Tetsuo F., Ono T., Kobayakawa T., Shimoyama Y., Kimura S., Nakayama K. (2014). Identification and characterization of prokaryotic dipeptidyl-peptidase 5 from *Porphyromonas gingivalis*. J. Biol. Chem..

[9] Aemaimanan P., Sattayasai N., Wara-Aswapati N., Pitiphat W., Suwannarong W., Prajaneh S., Taweechaisupapong S. (2009). Alanine aminopeptidase and dipeptidyl peptidase IV in saliva of chronic periodontitis patients. J. Periodontol..

[10] Rea D., Van Elzen R., De Winter H., Van Goethem S., Landuyt B., Luyten W., Schoofs L., Van Der Veken P., Augustyns K., De Meester I. (2017). Crystal structure of *Porphyromonas gingivalis* dipeptidyl peptidase 4 and structure-activity relationships based on inhibitor profiling. Eur. J. Med. Chem..

[11] Ohara-Nemoto Y., Shimoyama Y., Nakasato M., Nishimata H., Ishikawa T., Sasaki M., Kimura S., Nemoto T.K. (2018). Distribution of dipeptidyl peptidase (DPP) 4, DPP5, DPP7 and DPP11 in human oral microbiota-potent biomarkers indicating presence of periodontopathic bacteria. FEMS Microbiol. Lett..

[12] Nemoto T.K., Ohara-Nemoto Y. (2016). Exopeptidases and gingipains in *Porphyromonas gingivalis* as prerequisites for its amino acid metabolism. Jpn. Dent. Sci. Rev..

[13] Cordero O.J., Varela-Calviño R., López-González T., Grujic M., Juranic Z., Mouriño C., Hernández-Rodríguez Í., Rodríguez-López M., de la Iglesia B.A., Pego-Reigosa J.M. (2017). Anti-CD26 autoantibodies are involved in rheumatoid arthritis and show potential clinical interest. Clin. Biochem..

[14] Ohara-Nemoto Y., Nakasato M., Shimoyama Y., Baba T.T., Kobayakawa T., Ono T., Yaegashi T., Kimura S., Nemoto T.K. (2017). Degradation of Incretins and Modulation of Blood Glucose Levels by Periodontopathic Bacterial Dipeptidyl Peptidase 4. Infect. Immun..

[15] Hajishengallis G. (2022). Interconnection of periodontal disease and comorbidities: Evidence, mechanisms, and implications. Periodontology.

[16] Mainas G., Ide M., Rizzo M., Magan-Fernandez A., Mesa F., Nibali L. (2022). Managing the Systemic Impact of Periodontitis. Medicina.

[17] Lalla E., Papapanou P.N. (2011). Diabetes mellitus and periodontitis: A tale of two common interrelated diseases. Nat. Rev. Endocrinol..

[18] Preshaw P.M., Alba A.L., Herrera D., Jepsen S., Konstantinidis A., Makrilakis K., Taylor R. (2012). Periodontitis and diabetes: A two-way relationship. Diabetologia.

[19] Sanz M., Ceriello A., Buysschaert M., Chapple I., Demmer R.T., Graziani F., Herrera D., Jepsen S., Lione L., Madianos P. (2018). Scientific evidence on the links between periodontal diseases and diabetes: Consensus report and guidelines of the joint workshop on periodontal diseases and diabetes by the International diabetes Federation and the European Federation of Periodontology. Diabetes Res. Clin. Pract..

[20] Mesa F., Magan-Fernandez A., Castellino G., Chianetta R., Nibali L., Rizzo M. (2019). Periodontitis and mechanisms of cardiometabolic risk: Novel insights and future perspectives. Biochim. Biophys. Acta Mol. Basis Dis..

[21] Suzuki A., Ji G., Numabe Y., Ishii K., Muramatsu M., Kamoi K. (2004). Large-scale investigation of genomic markers for severe periodontitis. Odontology.

[22] Eley B.M., Cox S.W. (1995). Correlation between gingival crevicular fluid dipeptidyl peptidase II and IV activity and periodontal attachment loss. A 2-year longitudinal study in chronic periodontitis patients. Oral Dis..

[23] Beighton D., Life J.S. (1989). Trypsin-like, chymotrypsin-like and glycylprolyl dipeptidase activities in gingival crevicular fluid from human periodontal sites with gingivitis. Arch. Oral Biol..

[24] Eley B.M., Cox S.W. (1992). Cathepsin B/L-, elastase-, tryptase-,trypsin- and dipeptidyl peptidase IV-like activities in gingival crevicular fluid: Correlation with clinical parameters in untreated chronic periodontitis patients. J. Periodontal. Res..

[25] Jiang Y., Brandt B.W., Buijs M.J., Cheng L., Exterkate R.A.M., Crielaard W., Deng D.M. (2021). Manipulation of Saliva-Derived Microcosm Biofilms to Resemble Dysbiotic Subgingival Microbiota. Appl. Environ. Microbiol..

[26] Moraes R.M., Lima G.M., Oliveira F.E., Brito A.C., Pereira R.C., Oliveira L.D., Barros P.P., Franco G.C., Anbinder A.L. (2015). Exenatide and Sitagliptin Decrease Interleukin 1β, Matrix Metalloproteinase 9, and Nitric Oxide Synthase 2 Gene Expression but Does Not Reduce Alveolar Bone Loss in Rats with Periodontitis. J. Periodontol..

[27] Kumagai Y., Konishi K., Gomi T., Yagishita H., Yajima A., Yoshikawa M. (2000). Enzymatic properties of dipeptidyl aminopeptidase IV produced by the periodontal pathogen *Porphyromonas gingivalis* and its participation in virulence. Infect. Immun..

[28] Shimoyama Y., Sasaki D., Ohara-Nemoto Y., Nemoto T.K., Nakasato M., Sasaki M., Ishikawa T. (2023). Immunoelectron Microscopic Analysis of Dipeptidyl-Peptidases and Dipeptide Transporter Involved in Nutrient Acquisition in *Porphyromonas gingivalis*. Curr. Microbiol..

[29] Mohamed H.G., Idris S.B., Mustafa M., Ahmed M.F., Åstrøm A.N., Mustafa K., Ibrahim S.O. (2015). Impact of Chronic Periodontitis on Levels of Glucoregulatory Biomarkers in Gingival Crevicular Fluid of Adults with and without Type 2 Diabetes. PLoS ONE.

[30] Suvan J., Masi S., Harrington Z., Santini E., Raggi F., D’Aiuto F., Solini A. (2021). Effect of Treatment of Periodontitis on Incretin Axis in Obese and Nonobese Individuals: A Cohort Study. J. Clin. Endocrinol. Metab..

[31] Solini A., Suvan J., Santini E., Gennai S., Seghieri M., Masi S., Petrini M., D’Aiuto F., Graziani F. (2019). Periodontitis affects glucoregulatory hormones in severely obese individuals. Int. J. Obes..

[32] Wang Z., Wang X., Zhang L., Wang B., Xu B., Zhang J. (2020). GLP-1 inhibits PKCβ2 phosphorylation to improve the osteogenic differentiation potential of hPDLSCs in the AGE microenvironment. J. Diabetes Its Complicat..

[33] Guo Z., Chen R., Zhang F., Ding M., Wang P. (2018). Exendin-4 relieves the inhibitory effects of high glucose on the proliferation and osteoblastic differentiation of periodontal ligament stem cells. Arch. Oral Biol..

[34] Liang Q.Y., Du L.Q., Zhang R., Ding T., Ge S.H. (2020). Effects of Exenatide-4 on proliferation, migration and osteogenic differentiation of human periodontal ligament stem cells. Shanghai J. Stomatol..

[35] Ma X., Meng J., Jia M., Bi L., Zhou Y., Wang Y., Hu J., He G., Luo X. (2013). Exendin-4, a glucagon-like peptide-1 receptor agonist, prevents osteopenia by promoting bone formation and suppressing bone resorption in aged ovariectomized rats. J. Bone Miner. Res. Off. J. Am. Soc. Bone Miner. Res..

[36] Wang M., Liu M., Zheng J., Xiong L., Wang P. (2023). Exendin-4 regulates the MAPK and WNT signaling pathways to alleviate the osteogenic inhibition of periodontal ligament stem cells in a high glucose environment. Open Med..

[37] Sawada N., Adachi K., Nakamura N., Miyabe M., Ito M., Kobayashi S., Miyajima S.I., Suzuki Y., Kikuchi T., Mizutani M. (2020). Glucagon-Like Peptide-1 Receptor Agonist Liraglutide Ameliorates the Development of Periodontitis. J. Diabetes Res..

[38] Pang Y., Yuan X., Guo J., Wang X., Yang M., Zhu J., Wang J. (2019). The effect of liraglutide on the proliferation, migration, and osteogenic differentiation of human periodontal ligament cells. J. Periodontal Res..

[39] Zhang Y., Yuan X., Wu Y., Pei M., Yang M., Wu X., Pang Y., Wang J. (2020). Liraglutide regulates bone destruction and exhibits anti-inflammatory effects in periodontitis in vitro and in vivo. J. Dent..

[40] Zhai S., Liu C., Vimalraj S., Subramanian R., Abullais S.S., Arora S., Saravanan S. (2023). Glucagon-like peptide-1 receptor promotes osteoblast differentiation of dental pulp stem cells and bone formation in a zebrafish scale regeneration model. Peptides.

[41] Yang M., Pang Y., Pei M., Li Y., Yuan X., Tang R., Wang J. (2022). Therapeutic Potential of Liraglutide for Diabetes-Periodontitis Comorbidity: Killing Two Birds with One Stone. J. Diabetes Res..

